# Holocene Nile dynamics shaped the physical and cultural landscape of ancient Nubia

**DOI:** 10.1073/pnas.2529986123

**Published:** 2026-04-27

**Authors:** Jan Peeters, Timotheus G. Winkels, Pawel Wolf, Tim B. B. Skuldbøl, Elizabeth L. Chamberlain, Saskia Büchner-Matthews, Sami M. Elamin, El-Hassan A. Mohamed, Geoff Emberling

**Affiliations:** ^a^Jebel Barkal Archaeological Project, Kelsey Museum of Archaeology, College of Literature, Science, and the Arts, University of Michigan, Ann Arbor, MI 48109; ^b^Water & Environment Group, Transport and Infrastructure, William Sale Partnership, Nieuwegein 3439LM, The Netherlands; ^c^House on the Hill—A Danish Archaeological Research and Consultancy Unit, Glesborg 8585, Denmark; ^d^Netherlands Centre for Luminescence dating, Wageningen University, Wageningen 6700AA, The Netherlands; ^e^Soil Geography & Landscape Group, Soil Science cluster, Wageningen University, Wageningen 6700AA, The Netherlands; ^f^National Corporation for Antiquities and Museums, Khartoum 11111, Sudan; ^g^Kelsey Museum of Archaeology, College of Literature, Science, and the Arts, University of Michigan, Ann Arbor, MI 48109

**Keywords:** Sudan, Napata, Jebel Barkal, geomorphology, archaeology

## Abstract

The Nile River played a central role in ancient civilizations of Northeast Africa, yet its response to Holocene climate change and its impact on societies along its course remain poorly understood. Here we show how climatic and environmental changes over the past 12,500 y shaped the riverine landscape below the Nile’s Fourth Cataract and affected the Nubian empire of Kush in northern Sudan. Using 26 sediment cores dated by optically stimulated luminescence and radiocarbon, with additional chronological constraints from pottery typology, we reconstruct the evolution of the Nile near Napata, the major urban center of ancient Kush at Jebel Barkal (near present-day Karima). Napata, renowned for its pyramids, temples, and palaces, flourished from about 1070 BCE to 350 CE and is now a UNESCO World Heritage site. Our results reveal that the Early–Middle Holocene Nile deeply incised its valley, followed by widespread floodplain buildup around 4,000 y ago as rainfall and river flow patterns changed. During the Late Holocene, the Nile River near Jebel Barkal remained remarkably stable, due to its narrow valley with constrained outflow and enhanced sediment deposition from upstream energy dissipation at the cataract. This long-term stability promoted fertile floodplain development and, together with the sacred prominence of Jebel Barkal, fostered enduring settlement and ritual activity. These findings demonstrate how hydroclimatic change, geomorphic stability, and cultural adaptation were intertwined in shaping the environmental foundation of ancient Nubia.

## Significance

The ancient city of Napata at Jebel Barkal—home to pyramids, temples, and palaces and now a UNESCO World Heritage site—was a major urban center of the Nubian empire of Kush for over a millennium (1070 BCE to 350 CE). Despite extensive archaeological research, the surrounding Nile valley remains poorly understood, leaving human–environment interactions largely speculative. This geomorphological survey reveals that Holocene Nile sediments and local wadis created the stable, fertile landscape on which Napata was built. Integrating geological and archaeological evidence shows that shifts in Nile flow and sedimentation directly influenced where and how people settled. The results highlight how environmental change shaped the rise and persistence of one of Africa’s earliest riverine civilizations.

The Nile River—the longest river in the world (1)—played a fundamental role in the emergence of ancient Northeast African cultures (2, 3), including ancient Kush (4). Understanding the Nile’s fluvial dynamics and their impacts is therefore crucial, particularly in the context of the dramatic hydroclimatic shift from the “Green Sahara” of the North African Humid Period (c. 14.5 to 5.0 ka) (5, 6) to the present-day hyperarid Sahara Desert (7, 8).

Yet, despite the Nile’s central role in ancient African history, recent environmental research has largely focused on the Sahara Desert regions (2, 3, 9, 10) and the river’s megascale hydrographic basin (6, 11–15). As a result, detailed, site-specific knowledge that is required to understand the positioning and functioning of key archaeological sites along the Nile valley, such as Napata—including its influence on settlement patterns, floodplain dynamics, and cultural and economic resilience—remains limited (16).

**Fig. 1.**
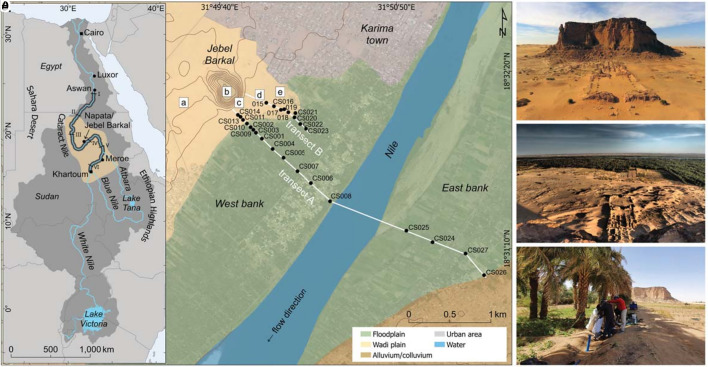
Overview of the Jebel Barkal area in Sudan. (*A*) Simplified geomorphic map of the Nile valley bordering Jebel Barkal (ancient Napata). Black dots mark core-site locations along transects A and B (white lines). Key archaeological sites (a–e): Royal Pyramids (a), Jebel Barkal (b), Great Amun Temple (c), Meroitic Palace (d), and the East Mound settlement area (e). The inset map shows the Nile Basin in Northeast Africa (dark gray), its drainage system (blue), the Cataract Nile (black shading), and the realm of Kush around 600 BCE (light brown). Nile Cataracts I–VI are indicated by capital Roman numerals. (*B*) Jebel Barkal (c. 105 m high) with its landmark pinnacle (c. 75 m high) at its far left, and the Great Amun Temple in front (looking in NW direction). (*C*) Overlooking the Great Amun Temple and East Mound settlement area with the Nile River (approximately 1 km away) and its floodplain with date-palm groves in the background (looking in SE direction). (*D*) Sediment drilling with percussion corer at CS001 with Jebel Barkal in the background. Credits: (*A*) background WorldView-3 satellite imagery © 2025 Maxar Technologies; (*B*) Sami Elamin; (*C*) Henrik Brahe; (*D*) Pawel Wolf.

For over two millennia (2000 BCE to 350 CE), Kush was centered along the Cataract Nile (Fig. 1*A*) and was one of the earliest and most influential civilizations in ancient Africa, rivaled only by ancient Egypt. In this paper, we present the findings of our interdisciplinary research into the Nile’s fluvial evolution at the archaeological site of Jebel Barkal: the ancient urban center of Kush, Napata (Fig. 1 *A*–*D*). Situated at the base of this prominent Nubian Sandstone inselberg (Fig. 1*B*), Napata was originally founded as an Egyptian fortified outpost during the New Kingdom in the early to mid-15th century BCE (17, 18). It became the principal urban center of the Kushite dynasty during much of the Napatan period (1070 to 300 BCE) and remained an important city during the subsequent Meroitic period (300 BCE to 350 CE) (16, 18).

Archaeological studies of Kush have occasionally included geomorphological perspectives, for example at Napata (16) and at Meroe/Hamadab (19, 20). Other research has instead focused on the geomorphology of the Fourth Cataract (21) immediately upstream of Jebel Barkal and the Northern Dongola Region (13, 22, 23), some 300 km downstream. The direct relationship between the ancient city of Napata and the Nile, along with the broader environmental dynamics of its immediate setting, remains however largely unexplored (16, 24).

**Fig. 2.**
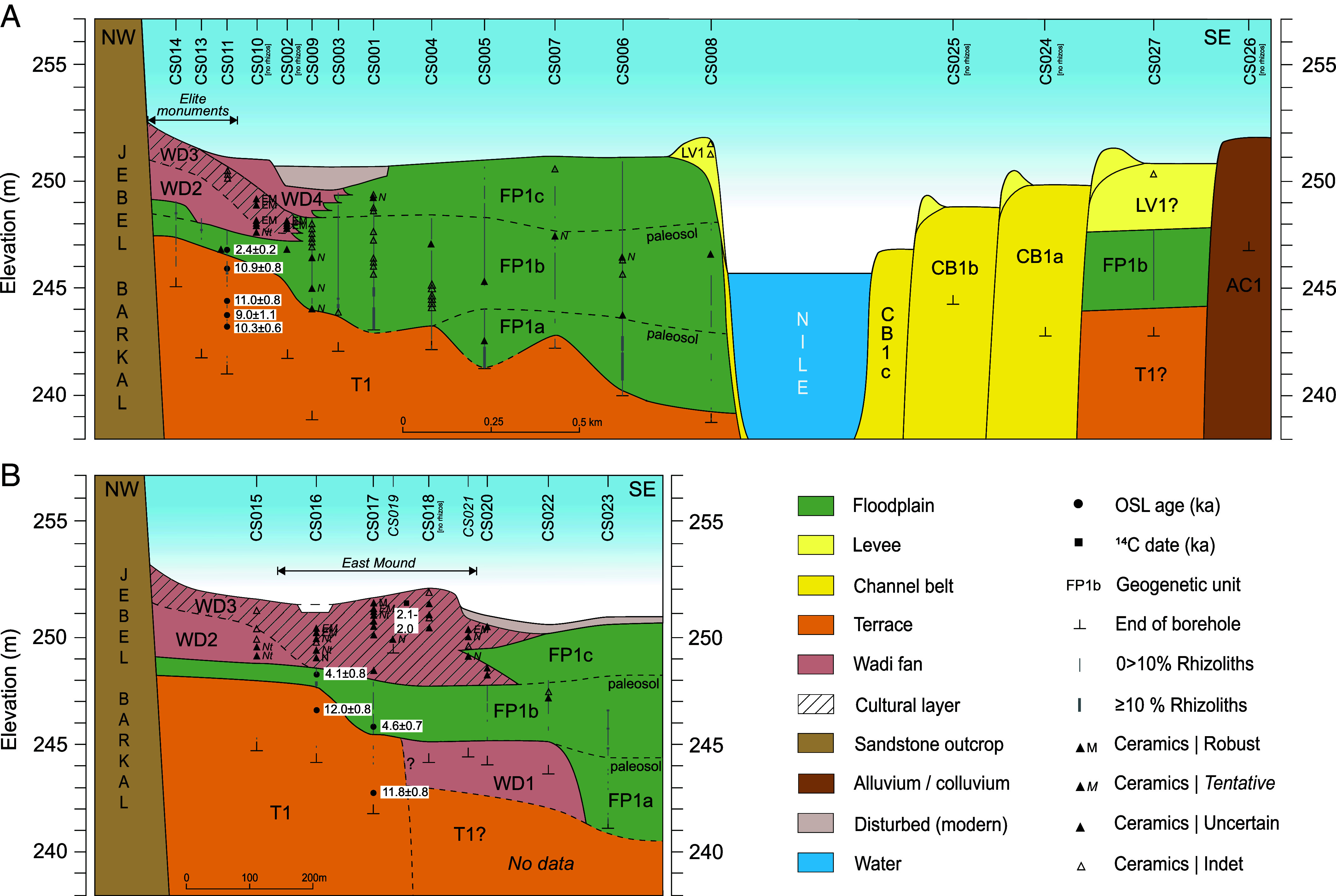
Sedimentary architecture of the Nile valley at Jebel Barkal. (*A*) Transect A, spanning the entire Nile valley; (*B*) transect B, detailing the East Mound settlement area. See Table 1 for descriptions of the geogenetic units. Note the difference in horizontal scale between transects A and B. Cores CS019 and CS021 are projected; see Fig. 1*A* for transect and core-site locations. Ceramic field-analysis archaeological period indications: Meroitic (M); Early Meroitic (EM); Napatan or transition to Meroitic (Nt); Napatan (N). OSL ages (1-σ uncertainty) and the calibrated ^14^C date (2-σ uncertainty) are presented as a range in ka relative to 2022, the first year of OSL sample collection. Values are shown to only one decimal place for legibility. No rhizoliths were found in cores CS002, CS010, CS018, CS024, CS025, or CS026. See *Materials and Methods* and *SI Appendix* for more details on the sedimentary data and interpretation, OSL and radiocarbon dating, and ceramic field-analysis.

To address this gap and to develop a more holistic understanding of the cultural adaptation and environmental resilience in ancient Napata, we analyzed two parallel drilled transects (c. 400 m apart; Fig. 1*A*): one spanning the full width of the Nile valley (c. 3,500 m wide; Fig. 2*A*), and the other focusing on the ancient settlement site (i.e., the East Mound; Figs. 1*C* and 2*B*) and its proximity to the river. Sedimentary data from 26 cores (average depth c. 8 m; see Dataset S1) were analyzed to reconstruct key events and transformations in the evolving landscape. These were temporally constrained by nine optically stimulated luminescence (OSL) ages, two radiocarbon dates (16), and corroborated by ceramic typology dating (see *SI Appendix* for details). This approach (25–28) offers a unique perspective on the Holocene evolution of the riverine environment at Jebel Barkal, the setting in which the ancient Kushite culture developed, thrived, and ultimately declined.

## Results

### Sedimentary Architecture of the Holocene Nile at Jebel Barkal.

The sedimentary record at Jebel Barkal shows a dynamic Holocene history of Nile incision and aggradation that shaped the geomorphic foundation of ancient Napata. Subsurface data from two transects (Fig. 2 *A* and *B*) reveal distinct sedimentary units that document this evolution, including Early–Middle Holocene terraces, Late Holocene floodplain and channel-belt deposits, and wadi-fan accumulations at the base of the inselberg.

Basal sandy deposits at various depths are interpreted as a fluvial–terrace complex (T1; Table 1), formed by valley-wide incision and channel contraction during the Early and Middle Holocene, a process likely initiated in the Late Pleistocene. This erosional phase was followed by Late Holocene aggradation, when the Nile infilled its valley and buried the terrace morphology. The resulting deposits include a single channel belt (CB1) comprising three side bars (CB1a–c), with associated natural levees (LV1) on both banks of the river and a laterally expanding, vertically aggrading floodplain (FP1a–c), marking renewed fluvial dynamics. At the northwestern valley margin near Jebel Barkal, laterally interfingering wadi-fan lobes (WD2–4) overlie parts of the floodplain, reflecting episodic input from ephemeral tributaries active during the Late Holocene.

**Table 1.** Summary of geogenetic units in the Holocene Nile valley at Jebel Barkal

Geogenetic unit	West/East Bank	Dominant texture(s)^*^, sedimentary structures	Facies interpretation	Age^†^
CB1(a-c)	EB	Cross-bedded, well-sorted sand (150 to 300 µm), with high mica content^‡^, mm- and cm-scale laminations, occasionally with some clay balls. Fining upward into somewhat heterogeneous textures: loamy sand, sandy loam, silt loam, silt, silty clay loam (ranging from 30 to 100 µm); all with a lower mica content.	Channel belt comprising three side bars; presumed riverbed incision of 1 to 2 m since 2008.	1960s to present-day Nile River.
LV1	WB + EB	Rapidly alternating (dm-scale), moderately to well-sorted, mica-poor to mica-rich, silt loam (60 to 75 µm) to fine-grained sand (100 to 200 µm). Fringing present or past young Nile channels.	Levee (natural); built-up by repetitive Nilotic floods.	1960s to present.
FP1a-c	WB + EB	Homogeneous very fine-grained (20 to 70 µm), moderate to well-sorted silty clay to silt loam, locally interbedded with fine- to medium-grained loamy sand or sandy loam. Calcareous rhizoliths occur throughout parts of the floodplain succession, associated with weakly developed paleosols.	Floodplain (three stages); Nilotic overbank deposits with weakly-developed paleosols.	FP1c: Late Holocene, <3.70 ± 0.61 ka to present; FP1b: 3.70 ± 0.61 ka (OSL age^§^); FP1a: Late Holocene, >3.70 ± 0.61 ka.
T1	WB + EB?	Stacked fining-upward sequences (1 to 3 m thick) with poorly to well sorted, mica-rich very fine- to coarse grained sand (75 to 1000 µm), often showing cm- and mm-laminations and 1 to 4 mm-thick clay drapes. Occasionally with fine- to coarse-sized quartz gravel towards the base of the sets.	Fluvial terrace complex consisting of short, stacked channel sets; possibly residual channel and reworked wadi material; possibly reworked top.^¶^	Early–Middle Holocene: 10.83 ± 0.81 ka (OSL age) and younger.
WD4	WB	Moderately to very poorly sorted, (loamy) sands (250 to 450 µm), often containing fine- to medium sized (5 to 20 mm) gravel (5 to 10%). No micas.	Reworked (disturbed?) wadi fan material, presumably mixed with nearby eolian material.	(Sub-)modern.
WD3/Cultural layer	WB	Poorly to very poorly sorted, fine- to medium grained (80 to 450 µm) (sandy) loam and (loamy) sands, often containing medium sized (5 to 20 mm) gravel (5 to 10%). Occasionally silt loam (40 to 75 µm). Little to no micas. Often containing cultural material: ceramic sherds, bone fragments, flint flakes, charcoal. Highly variable, heterogenous in texture occurrence.	Wadi-fan lobe with urban structural remains and areas of miscellaneous anthropogenic activity.	Napatan to Meroitic: c. 1070 BCE to 350 CE/3.1 to 1.7 ka. Top of East Mound: 2.1 to 2.0 ka (^14^C age^#^).
WD2	WB	Very poorly sorted, angular (loamy) sands (100 to 1,000 µm), often containing medium- to coarse sized (2 to 5 cm) gravel (10 to 30%). No micas.	Wadi-fan lobe.	Napatan: c. 1070 to 300 BCE/3.1 to 2.3 ka.
WD1	WB	Coarse, well-rounded quartz gravel (2 to 5 cm), in a matrix of very poorly sorted, coarse to very coarse sands (450 to 2,000 µm). No micas. The gravel clasts form the residue of the eroded basal Nubian Sandstone pebble conglomerate.	Wadi (fan), entering the Nile valley.	Middle Holocene (presumably): >3.70 ± 0.61 ka to 10.83 ± 0.81 ka.
AC1	EB	Somewhat consolidated rounded to subrounded gravel of polygenetic origin in a matrix of coarse sand/Cross-bedded fluvial sand with minor conglomerate and clay layers.	Alluvium/Colluvium; deposited by ephemeral streams.	Pleistocene (Prenile).

^*^Sedimentary texture descriptions conform to United States Department of Agriculture standards (46).

^†^Ages are based on OSL dating, radiocarbon dating, ceramic typology, stratigraphic relationships, and CORONA satellite imagery; see *SI Appendix*, SI Text for details.

^‡^The presence of mica is typically indicative of Nile-derived sediment input from upstream metamorphic sources and helps distinguish Nilotic deposits from locally derived wadi sediments.

^§^The OSL ages presented here represent the median OSL age per geogenetic unit; see *SI Appendix*, SI Text for details.

^¶^Due to its deeply buried position (9 to 12 m beneath the surface), core penetration of the lower part of T1 was limited to <1 m.

^#^The age presented here represents the weighted mean calibrated ^14^C age for the top of the East Mound; see *SI Appendix*, SI Text for calibration details.

The oldest sections of the terrace complex (T1; Fig. 2 *A* and *B* and Table 1) date to the Early Holocene (OSL age: 10.83 ± 0.81 ka) and flank Jebel Barkal at c. 247 m elevation. Eastward, the terrace surface slopes 2 to 3 m down to c. 245 m. A distal remnant of T1 appears also to be preserved near the desert edge on the East Bank (AC1; Fig. 2*A* and Table 1). Toward the valley center, the youngest terrace section occurs at 239 to 243 m and gently slopes toward the center of the valley. In general, the fluvial–terrace complex consists of short, stacked sequences that are consistent with dynamic channel deposition. Due to deep burial under thick Holocene floodplain deposits, only a broad Early–Middle Holocene age could be assigned here. This part of unit T1 forms the substrate from which fluvial aggradation began, marking a key transition in the local Nile’s history.

Adjacent to the upper T1 terrace section, a gravel-rich deposit appears to cut into the lower section of the terrace and is interpreted as the terminus of a wadi fan (WD1; Fig. 2*B* and Table 1), likely entering the Nile valley from upstream near Karima (Fig. 1*A*). Although its surface lies at a similar elevation (c. 245 m) to the middle section of T1, the sediment composition is notably different. Its position suggests deposition predating the main phase of Late Holocene floodplain aggradation.

Floodplain deposits (FP1a–c; Fig. 2 *A* and *B* and Table 1) blanket large parts of both Nile banks, ranging from c. 1 m thick in the far northwest to c. 10.5 m near the present river. A weakly developed paleosol marks the transition from FP1a to FP1b, the most extensive floodplain unit (OSL age: 3.70 ± 0.61 ka). Because FP1a underlies this dated unit, sedimentation must have begun prior to 3.70 ± 0.61 ka, most likely at the onset of the Late Holocene. The paleosols separating FP1a–c are weakly developed, characterized by slight structure development, localized carbonate accumulation, and abundant calcareous rhizoliths, indicating short-lived reductions in sedimentation rather than prolonged landscape stability. Another paleosol separates FP1b from FP1c, the most recent Nilotic overbank deposits. Ceramic fragments are found throughout large parts of the floodplain and are most concentrated in unit FP1b (Fig. 2*A*). Overlying these deposits are the Nile’s natural levees (LV1; Fig. 2*A* and Table 1), which form prominent ridges at c. 251.5 m in the valley center.

A single, active channel belt (CB1) is identified on the East Bank (Fig. 2*A* and Table 1), within which three side bars (CB1a–c) are distinguished. The bars are 100 to 250 m wide and 1968 CE or younger in age based on CORONA satellite imagery (see *SI Appendix* for details). The absence of soil development or rhizoliths indicates very recent formation. Since the commissioning of the Merowe Dam just upstream at the Fourth Cataract in 2008–2009, the river has likely shifted from limited lateral migration within its active channel belt toward enhanced vertical incision, consistent with reduced sediment supply.

At least three distinct wadi-fan lobes (WD2–4; Table 1) overlie the floodplain deposits near the base of Jebel Barkal (Fig. 2 *A* and *B*), displaying sediments which were washed down from the bordering wadi plain. WD2 (dated to c. 3.1 to 2.3 ka) is likely the distal extent of a major fan rounding the Jebel from the west and appears to cut into older floodplain sediments. WD3 (dating to c. 3.1 to 1.7 ka) originates from the north and contains abundant cultural material, including ceramic sherds, bone fragments, charcoal, and flint flakes, forming the main cultural layer of Jebel Barkal. This layer includes the slightly elevated East Mound settlement area (Fig. 1 *A* and *C*), with top deposits dated to 2.1 to 2.0 ka and currently under archaeological study (16, 29). WD4, which is limited in extent, represents (sub-)recently reworked fan material, mixed with local aeolian sediments.

Together, these sedimentary successions record the transition from an incised Early–Middle Holocene valley to a dynamically aggrading Late Holocene floodplain and wadi-fan complex, establishing the fluvial and geomorphic framework within which Napata developed.

## Discussion

### Hydroclimatic Impact on the Cataract Nile’s System Dynamics.

**Fig. 3.**
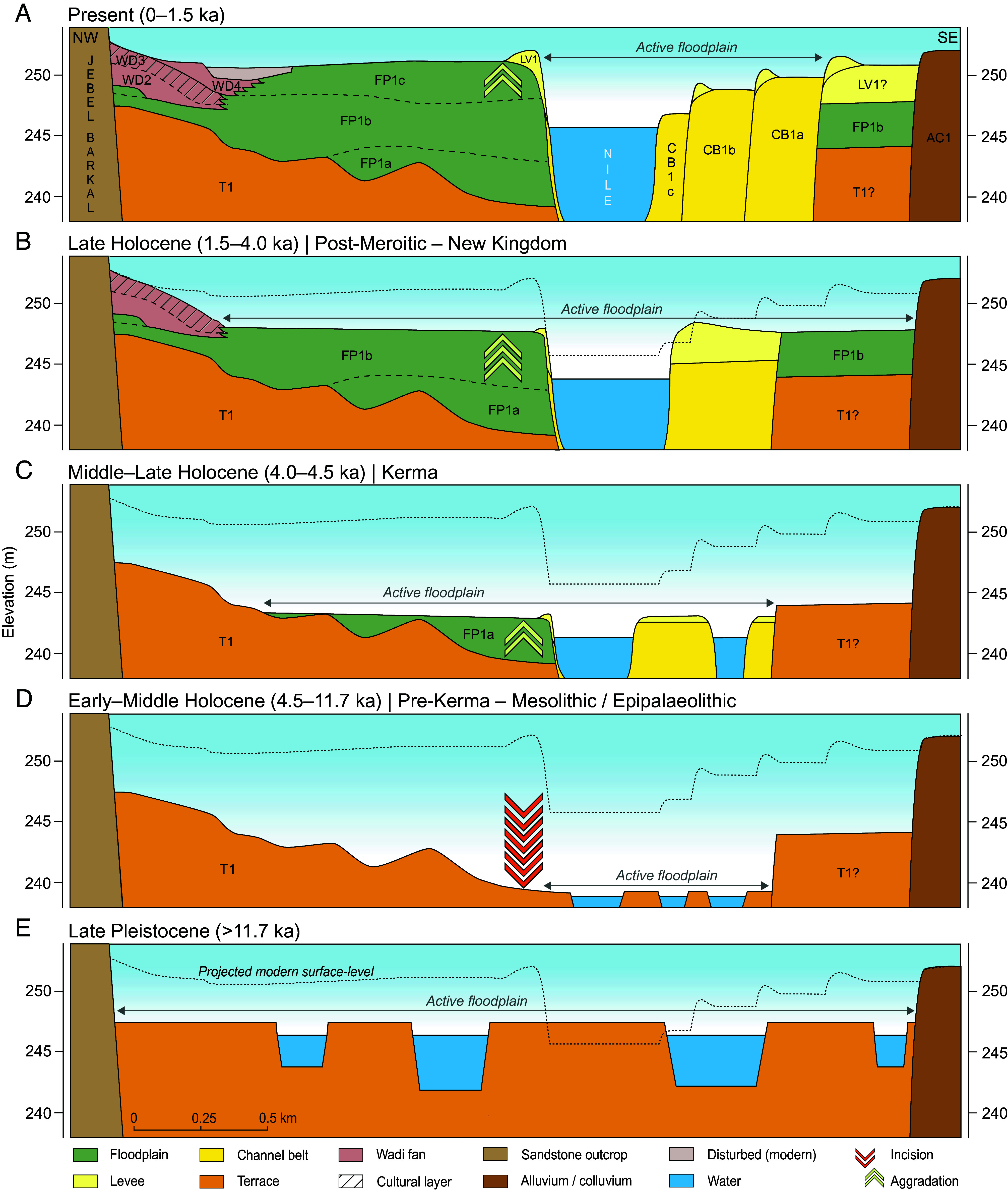
Schematic reconstruction of the Holocene evolution of the Nile valley at Jebel Barkal. Panels (*A*–*E*) are arranged from the present landscape (*Top*) to the Late Pleistocene (*Bottom*) but are described in chronological order (*E* = oldest; *A* = youngest). From the Late Pleistocene–Early Holocene onward (*E*), the Nile River eroded large parts of its older deposits (max. 9 m), creating a terraced landscape (T1), with multiple active channels in an ever-narrowing floodplain (*D* and *E*). Around the onset of the Late Holocene (*C*), the Nile started to aggrade (FP1a–c; max. 12 m) while building up its single channel belt in a consistently widening floodplain (*A–C*), with successive side bars (CB1a–c) representing reworking within the active belt. The difference between the projected modern surface-level and the respective paleo surface-levels indicates the amount of erosion or accumulation between these periods. Cultural periods at Jebel Barkal: see *SI Appendix*, Table S4. Note: time slices are for visual grouping and do not imply synchronous boundaries between geomorphic phases and archaeological periods.

**Fig. 4.**
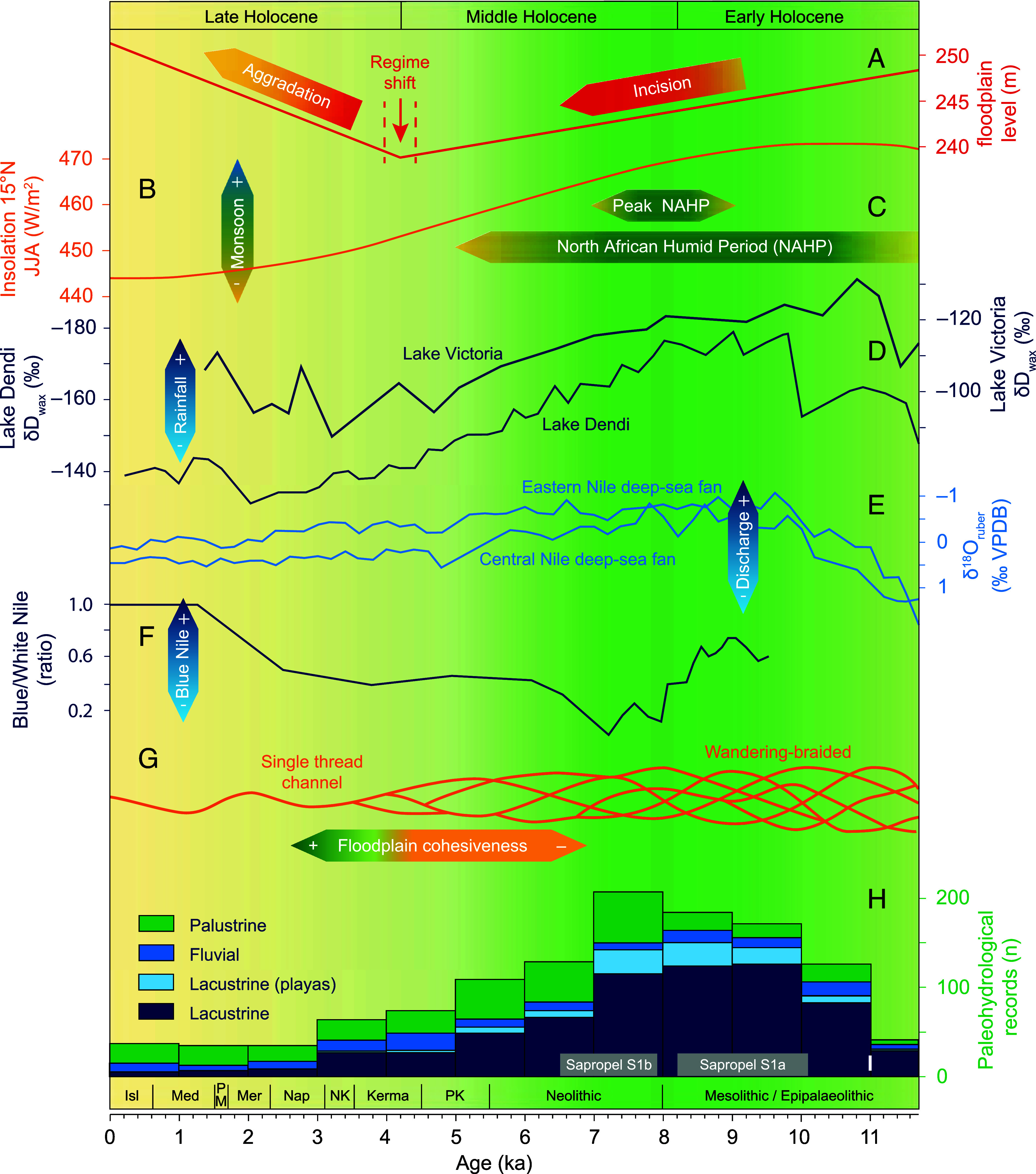
Fluvial evolution of the Cataract Nile at Jebel Barkal (Sudan) in relation to hydroclimatic changes in the Nile Basin. (*A*) Schematic Cataract Nile valley floodplain incision and aggradation levels (this study). (*B*) Summer insolation (June, July, August) at 15 °N (34). (*C*) North African Humid Period duration (NAHP) (5) and peak NAHP (6). (*D*) Precipitation variability inferred from leaf wax (δD_wax_) in the Ethiopian Highlands (Lake Dendi) (36) and the Lake Victoria basin (35). (*E*) Nile discharge signal from *Globigerinoides ruber* oxygen isotopes (12). (*F*) Relative Blue Nile discharge intensity (11). (*G*) Planform of the Nile River at Jebel Barkal (this study). (*H*) Humidification and aridification of the Sahara and Sahel recorded in North African paleohydrological lake archives (10). (*I*) Eastern Mediterranean Sapropel S1a/b (48). Cultural periods at Jebel Barkal: *SI Appendix*, Table S4.

Our interdisciplinary research reveals previously unrecognized behavior in the staging, timing, and mechanisms of earlier Cataract Nile river models (13, 22, 30, 31) and adds to recent advances in understanding Holocene Nile system dynamics (25–27). We identified a major fluvial regime shift, marked by channel contraction and entrenchment (max. 9 m depth) during the Late Pleistocene to Early–Middle Holocene, followed by valley-wide aggradation (max. 12 m thickness) around the onset of the Late Holocene (c. 4 ka) (Figs. 3 *A*–*E* and 4*A*), potentially aligning with the 4.2 ka climate event (32, 33). Such large-scale system shifts are typically associated with changes in hydroclimatic regimes, which force increases in sediment supply and fining and/or reductions in discharge, as was recently attested downstream in the Egyptian Nile Valley (27). The record at Jebel Barkal thus extends the downstream evidence of Peeters et al. (27) into the Cataract Nile, suggesting that the Late Holocene transition represents a basin-wide fluvial response to the 4.2 ka event.

The changes observed at Jebel Barkal align with evidence of similar changes elsewhere in the Nile Basin and likely result from comparable drivers (Fig. 4 *B*–*I*). From the Early Holocene, astronomically forced insolation changes (Fig. 4*B*) (34) impacted monsoon dynamics, causing relatively wet conditions over northern Africa (Fig. 4 *D*, *E*, and *H*) (7, 8, 10, 35, 36), which increased the Nile’s discharge, transport capacity, and erosivity (Fig. 4*E*) (11, 12, 14). Wetter conditions also reduced upstream sediment input (Fig. 4*F*) (11, 12), as denser vegetation protected soils from erosion (7, 8). Observed valley erosion and associated sediment uptake were thus direct results of a wetter hydroclimatic regime. In contrast, rapid aggradation from c. 4 ka onward stemmed from diminishing discharge, reduced erosion and transport capacity, and increased fine sediment supply (11, 12, 23, 37). This shift was mainly driven by progressive aridification, especially between 5 and 6 ka (Fig. 4 *C* and *H*) (5, 7, 10), likely aided by increasing human impacts (2, 38, 39).

The shift from erosion to aggradation marked a turning point in the fluvial development of the Nile near Jebel Barkal, transforming the river from a dynamic, wandering-braided system with short, stacked channel sets (T1) into the present single-thread channel (CB1) (Figs. 3 *A*–*E* and 4*G*). Unlike the major Late Holocene reconfigurations observed elsewhere—such as near the Giza Plateau (40, 41), in Middle and Upper Egypt (25–27), or along the Dongola Reach (22)—the Nile’s channel belt position at Jebel Barkal remained remarkably stable in the center of its valley. And although the fluvial evolution near Jebel Barkal broadly resembles other Nile reaches characterized by overbank sedimentation, it contrasts with the markedly different dynamics observed in the Dongola Reach (22), highlighting that significant variability can exist even within a single reach between cataracts. In the case of Jebel Barkal, the proximity of the Fourth Cataract (<15 km upstream), a >150 km-long bedrock-controlled reach of the Nile (21), appears to have amplified aggradational processes, contributing to long-term geomorphic stability. Fine-grained sediment input likely promoted cohesive bank and floodplain development, increasing erosion resistance and facilitating progressive channel stabilization.

The Nile valley at Jebel Barkal is narrow, measuring only 3 km across. This contrasts with the broader floodplains elsewhere along the Nile: for example, in the Meroe region (4 to 8 km wide) (19), the Northern Dongola Reach (15 to 20 km wide) (22), and the Egyptian Nile Valley (10 to 20 km wide) (27, 30). This narrowness likely constrained lateral channel mobility by limiting the potential for large-scale avulsions, secondary channels, and complex anabranching systems, thereby favoring maintenance of a stable single channel belt. It may also explain the gentler terrace gradients observed in this area, which contrast with the more pronounced stepped sequences near Luxor (27). Additionally, the nearby Fourth Cataract (21) likely imposed a persistent upstream hydraulic boundary condition, moderating energy gradients and dampening channel instability. Together, valley confinement and cataract-induced boundary forcing created conditions conducive to sustained floodplain aggradation in this reach.

### Shaping the Ancient Landscape at Jebel Barkal.

The Nile valley at Jebel Barkal provides a unique record of Holocene fluvial evolution, allowing us to link hydroclimatic change directly to formation of the Kushite cultural landscape. Around the onset of the Late Holocene (c. 4 ka), floodplain aggradation at Jebel Barkal began and continued until the early 20th century CE, when upstream dam construction altered the Nile’s natural flood regime. At its thickest, approximately 11 m of overbank sediment accumulated near the valley axis (i.e., core CS006), yielding an average floodplain aggradation rate of c. 2.8 mm/year, a figure broadly consistent with Late Holocene sedimentation rates reported elsewhere along the Nile (25, 27, 40, 42).

The two weakly developed paleosols dividing the floodplain sequence into three depositional stages (FP1a–c), likely mark intervals of reduced sedimentation linked to diminished overbank flooding. While such slowdowns have traditionally been interpreted as periods of reduced Nile discharge (e.g., 1350 to 900 and 800 to 550 BCE) (13), recent work suggests that lower accumulation rates may also result from lateral floodplain expansion (27). In this scenario, previously elevated terrace surfaces became overtopped by fine-grained overbank deposits that were consequently spread thinly, such as during the transition from FP1a to FP1b (Fig. 2 *A* and *B*). From these intervals of reduced deposition, it naturally follows that sedimentation rates would have been higher during the intermediate phases of the Late Holocene aggradational sequence.

Wadis, recognized as major contributors to valley floor sedimentation along the Nile (23), were another important factor in shaping the landscape with their episodic sediment input. These ephemeral tributaries were active along the northwestern valley margin and in the plain surrounding the Jebel. Upon entering the lower-lying floodplain, they built a laterally interfingering wadi-fan system (WD2–3; Fig. 2 *A* and *B*), a characteristic feature of the Late Holocene landscape. This wadi-fan system comprises multiple lobes that were likely active both sequentially and at times simultaneously, creating a highly dynamic and complex sedimentary environment. Importantly, this highly heterogenic unit provided the foundation upon which the city of Napata was established: temples and palaces on the wadi plain at the foot of Jebel Barkal (Fig. 1 *A*–*C*), and the ancient settlement area a bit further away on the wadi-fan comprising the East Mound.

### Implications for the Kushite Cultural Landscape.

The geomorphic stability and landscape configuration reconstructed at Jebel Barkal formed the environmental backdrop to the rise of the Kushite empire. At the onset of the Napatan period (c. 1070 BCE), climate-driven aridification across the Eastern Sahara approached approximately present-day conditions (5, 9, 10) (Fig. 4 *C* and *H*), although local variations may still be documented. This growing aridity increasingly concentrated human settlement and activity in the narrow Nile corridor (2). Even within this corridor, conditions further deteriorated, with declining trends in Nile discharge (Fig. 4*E*) (12) and reduced flood magnitude and frequency (13) during the Middle and Late Holocene. Yet despite these challenges, the city of Napata emerged and grew into an imperial capital (16, 18, 21).

An apparent wet phase coincided with the Napatan period (13). Increased precipitation in the White Nile’s headwaters (Fig. 4*D*) temporarily produced higher flows and overbank flooding in the Northern Dongola Reach (22). Under otherwise desiccating conditions, such episodes likely also benefited other reaches of the Cataract Nile. At Jebel Barkal, these hydrological conditions coincided with steadily widening floodplains on both sides of the Nile (Fig. 3 *A*–*C*). Together, a stable and favorable fluvial regime and the newly formed fertile lands in all likelihood encouraged settlement. The sacred and topographic prominence of Jebel Barkal (17, 18), with occupation around its base since the Classic Kerma period (c. 1750 to 1550 BCE) (43), likely further guided the selection of Napata’s location.

Settlements along the Nile’s confined valley were likely established on elevated ground, set back from the flood-prone, aggrading valley floor to ensure some degree of flood security. At Napata, the site occupies the very edge of the Nile valley, with its East Mound settlement projecting approximately 300 m into the floodplain. Although about 1 km from the river channel, these elevated sites provided strategic access to the Nile for water, transport and trade, including interaction with Sanam (44), Napata’s major economic hub located c. 5 km downstream across the river. By positioning the settlements on naturally elevated ground while maintaining proximity to its riverine resources, Napata illustrates a deliberate adaptive strategy, mitigating flood risk while maximizing accessibility and connectivity. In this context, environmental change appears to have created not only constraints but also opportunities that were actively exploited in the development of Napata.

While Napata’s strategic location allowed the city to thrive under fluctuating hydrological conditions, these advantages depended on sufficient Nile flows, making the settlement ultimately sensitive to long-term climatic trends. Climate-induced desiccation may have contributed to the shift in the Kushite center of power from Napata to Meroe around 300 BCE (31). This political shift coincided with the end of the apparent wet Napatan period. The subsequent long-term reduction in Nile water availability (Fig. 4*E*), potentially driven by volcanically triggered hydroclimatic changes (45), would have severely affected Napata’s viability. Meroe, located approximately 250 km southeast of Napata in a seasonally rain-fed savannah environment (20), likely offered an environment with a greater diversity of subsistence strategies, reflecting a climate-adaptive relocation (20, 31).

Archaeological evidence from the Kushite period also reveals abrupt shifts in urban development that cannot be explained by the relatively gradual environmental changes of the Late Holocene (16, 18, 20). The shift in power from Napata to Meroe reflects a complex interplay of climatic, cultural, and sociopolitical factors. Environmental change, particularly shifts in hydroclimatic regimes and sedimentary dynamics, shaped the physical landscape of the Cataract Nile and conditioned the possibilities for settlement. Yet the trajectory of the Kushite state was not determined by environment alone: it was forged through human choices. Strategic site selection, adaptive land use, and shifting sociopolitical priorities that transformed challenges into opportunities, ultimately shaped the cultural landscape of ancient Kush. This integrated record illustrates the close relationship between human activity and river dynamics along the Nile during a key phase in North Africa’s cultural development.

## Materials and Methods

Sediment samples were retrieved using an Eijkelkamp hand auger and/or Cobra TT percussion corer (Fig. 1*D*) and their characteristics were logged on-site conforming to United States Department of Agriculture standards (46). OSL dating provided robust ages for the deposition of geomorphic units, which were corroborated by chronological information from the typological dating of ceramic fragments encountered in the drilled sediments. A weighted mean of 2 accelerator mass spectrometry (AMS) radiocarbon dates from the East Mound settlement area, recalibrated from Emberling et al. (16) with IntCal20 (47) was used to date the cultural layer (Fig. 2*B*). For more details, see *SI Appendix*, *Extended Materials and Methodology*.

## Supplementary Material

Appendix 01 (PDF)

Dataset S01 (XLSX)

## Data Availability

All study data are included in the article and/or supporting information.
