# Supplementary material for: Holocene Nile dynamics shaped the physical and cultural landscape of ancient Nubia
Source: Proc Natl Acad Sci U S A. 2026 Apr 27;123(18):e2529986123. doi: 10.1073/pnas.2529986123 (PMC13142976; doi:10.1073/pnas.2529986123)
Supplement: Supplementary file 1 — Appendix 01 (PDF) [file pnas.2529986123.sapp.pdf]

PNAS

Supporting Information for

Holocene Nile dynamics shaped the physical and cultural landscape of ancient Nubia

Jan Peeters*
Timotheus G. Winkels
Pawel Wolf
Tim B.B. Skuldbøl
Elizabeth L. Chamberlain
Saskia Büchner-Matthews
Sami M. Elamin
El-Hassan A. Mohamed
Geoff Emberling*

*To whom correspondence may be addressed: jpeeters@umich.edu or geoffe@umich.edu.

This PDF file includes:

Supporting text
Figures S1 to S6
Tables S1 to S5
SI References

Other supporting materials for this manuscript include the following:

Dataset S1

Supporting text

Extended Materials and Methodology

Sedimentary data and interpretation. Sedimentary information from 26 sediment cores was retrieved by a combination of hand-operated Eijkelkamp augers and a gasoline-powered Cobra TT percussion corer. Sediment samples were studied in 10 cm intervals and had their characteristics (conforming to United States Department of Agriculture (USDA) standards) (1) such as texture, grain size, color, degree of sorting, mica occurrence, ceramic-fragment occurrence and rhizolith percentages logged on-site. Boreholes reached an average depth of approximately 8 m — with many penetrating >10 m. Their spacing varied from 40 to 400 m, depending on the heterogeneity of the subsurface. Transect A was strategically placed to span the entire valley, perpendicular to the main axis of the Nile valley and the current river. Transect B was placed to cross the East Mound at the point of its maximum protrusion into the floodplain while staying parallel to transect A. Governmental policies and regulatory procedures for working in and around protected Sudanese Antiquities areas where followed. Coring locations were recorded in UTM Zone 36N (WGS84) using a handheld Garmin GPSMap 64s, with the vertical elevations surveyed using a Leica Total Station and dumpy level. For publication purposes, coordinates were converted to DMS. All location data was integrally stored with the sedimentary logs for future reference (see Dataset S1). The core run of CS012 was terminated early in compliance with regulatory requirements related to the encounter of antiquities. The sedimentary data collected from the top 1.4 meters was projected onto core CS014 (at approximately 4 m distance from CS012) to supplement its corresponding interval, which was unsuited for collection.

Robust age information was provided through 9 quartz optically stimulated luminescence (OSL) ages, originating from 3 core sites (i.e. CS011, CS016 and CS017) spread across the antiquities area, strategically targeting various sedimentary units (Fig. 2), for which OSL ages with 1σ standard deviation were calculated (Table 1; see below for further details on OSL dating). Chronology was further informed by radiocarbon dating of two charcoal samples from the East Mound cultural layer, previously reported by *Emberling et al.* (2). In addition, ceramic fragments found in the drilled sediments were analyzed and assigned to archaeological periods by typological dating (Table 1; see below for further details on ceramic analysis), aiding the sedimentary (chrono)stratigraphy.

OSL sampling and laboratory preparation. Based on the initial interpretation and reconstruction of the Holocene fluvial architecture in the Nile valley at Jebel Barkal, and after thorough inspection of the sedimentary logs, core-site locations were selected and revisited (within 1 m of their original borehole) to sample for OSL dating; see Table S1 for detailed sample locations and depths. OSL samples were collected using percussion coring equipment driven by a gasoline-powered Cobra TT hammer. For sampling, a metal core sampler (diameter 63 mm) with an exchangeable core catcher, lined with a dedicated black opaque thermoplastic tube was used to take undisturbed sediment samples of 100 cm in length. To prevent any possible disturbance, samples were favorably taken from homogeneous intervals and sampling across bounding surfaces was avoided. After the sample was lifted to the surface and extruded from the sampler, the liner containing the OSL sample was cut to length (30 cm), capped at both ends,

labelled, and wrapped in a non-transparent black plastic bag to avoid potential exposure to light. Sediment samples were subsequently transferred to the Netherlands Centre for Luminescence dating at Wageningen University & Research (The Netherlands) for dating under permit obtained by the Sudanese National Corporation of Antiquities and Museums (NCAM).

After transportation, samples were opened and prepared under subdued orange-light conditions, with the light-exposed sample ends removed to avoid contamination. Sediment preparation followed standard laboratory procedures (3). The grain-size fraction of 212–250 µm was obtained by wet sieving, and carbonates and organics were removed by chemical treatment with hydrochloric acid and hydrogen peroxide. The magnetic and non-magnetic mineral fractions of the purified samples were magnetic separated using a Frantz LB1 (4). Subsequent sodium polytungstate heavy liquid density separation at a density of 2.58 kg/cm^3 was performed to obtain quartz, which was afterwards etched with hydrofluoric acid to remove the alpha-irradiated outer layer of the quartz grains and rinsed with hydrochloric acid.

OSL dating procedures: Paleodose measurement and calculation. The OSL signal was obtained from the 212–250 µm purified quartz grains using the Single Aliquot Regenerative (SAR) dose protocol (5) (Table S2). The SAR protocol was performed on at least 22 aliquots per sample to obtain single-aliquot equivalent doses (D_e values). Each of these aliquots consisted of a sample disc containing approximately 30 grains (1-mm diameter samples of 212–250 µm purified quartz).

The OSL signal of quartz grains was integrated using the 'Early Background' approach (6). Aliquot acceptance criteria included a test dose error of less than 15%, recuperation less than 10% of the greatest regenerative dose and recycling ratios of less than 15% including one test for infrared (IR) depletion (7). With these criteria, 17–65% of aliquots were accepted for each sample, indicating that the small-aliquot approach sufficiently mimicked single-grain measurements.

Preliminary tests showed that quartz yielded a bright and suitable signal for OSL dating (Fig. S1). A suitable preheat temperature was verified with a thermal transfer test, that was conducted for samples CS016.B and CS017.B on eight 2-mm diameter aliquots (4 aliquots per sample) containing approximately 75 grains each (Fig. S2). Results showed increasing amounts of thermal transfer at temperatures greater than 240 °C, informing our selected preheat temperature of 220 °C. The SAR protocol was validated with a dose recovery test, which was performed on samples CS011.E, CS016.A, CS016.B and CS017.B, where a 25 Gy dose was given to 16 aliquots (4 aliquots per sample) of 2-mm diameter containing approximately 75 grains each. Eleven (69%) of the aliquots were accepted. The measured dose agreed favorably with the given dose (dose recovery ratio: 0.94 ± 0.02, n=11; Fig. S3).

Decisions regarding the statistical treatment of the single-aliquot D_e values, or 'age modelling', are critical to arriving at accurate paleodoses. We used the D_e distribution and its overdispersion value determined by the Central Age Model (Table S1) for each sample as a means to inform age model decisions.

Equivalent dose distributions measured on samples CS011.B, CS011.C, CS011.E, CS016.B and CS017.B (Fig. S4; Table S1) showed low scatter in the D_e values. For these samples, we first cleaned the aliquot datasets with a 2-standard deviation threshold, iteratively rejecting D_e values deviating more than 2 standard deviations from the sample mean. We then calculated the paleodose using an arithmetic mean and standard error. The iterated mean approach should

remove the D_e values of the youngest grains introduced by bioturbation and the oldest grains representing poorly bleached material if the populations are relatively minor.

Equivalent dose distributions of samples CS011.A, CS011.D, CS016.A and CS017.A (Fig. S4; Table S1) showed high scatter suggesting mixing of grains of different ages for example by bioturbation or agricultural practices, or incomplete resetting of the OSL signal in some grains prior to deposition (heterogeneous bleaching). For these samples, the source of scatter is thought to be most likely related to post-depositional bioturbation based on the lithology and environmental context of the sampled deposits as well as the cultural setting.

To obtain a representative paleodose, we again applied the iterated mean with a 2-standard-deviation threshold. We also applied the bootstrapped version (8) of the Minimum Age Model (9) ("bootMAM") to the full dataset of aliquots (meaning, including aliquots removed by the iterative approach). The iterated mean results are considered representative of the depositional age of the sampled sediments, and the bootMAM results can be considered minimum ages related to post depositional soil processes (i.e., when the deposits were last mixed, bioturbated, or otherwise cultivated to the sample depth). Both results are provided in the radial plots (Fig. S4). For sample CS011.A, we observed that the bootMAM result was strongly influenced by one near-zero D_e, yielding an underestimate of the paleodose. For this one sample, we manually excluded the one near-zero aliquot from age modelling.

The resulting iterated mean paleodoses of all samples were used for age calculation and are presented in Table S1 and in the radial plots (Fig. S4).

OSL dating procedures: Environmental dose rate determination. For environmental dose rate determination, radionuclide activities of surrounding sediment were obtained from wax-matrix and bulk sediment pucks measured on a gamma spectrometer. Resulting activity concentrations are presented in Table S1. We assumed gradual burial for all samples, including those of the terrace and floodplain deposits. This is because the terrace deposits were later buried by floodplain deposition, and the floodplain deposits were later buried by wadi fan deposition. It is likely that the water content of the deposits changed over time due to changes in burial depth, environment, and modern river management. The *in-situ* water contents at the time of sampling are provided in Table S1 and assumed values were used for dose rate calculation to approximate the average water content of the deposits over the duration of burial. We assumed time-averaged water contents of 15 ± 5% for deep terrace samples (CS011.C, CS011.D, CS011.E, and CS017.B). We assumed time-averaged water contents of 10 ± 5% for shallow terrace samples (CS011.B and CS016.B) and floodplain samples (CS011.A, CS016.A, and CS017.A). We included an internal alpha dose of 0.010 ± 0.005 Gy/ka in the dose rate calculation (10). Cosmogenic contributions were determined following *Prescott and Hutton* (11), grain-size attenuation was determined following *Mejdahl* (12) and attenuation by water content and organics was determined following *Aitken* (13). There were no signs of disequilibrium in the Uranium decay chain. Resulting dose rate values range from 1.64 ± 0.08 to 2.16 ± 0.11 Gy/ka, which is reasonable for Nile river alluvium (14).

OSL dating results. For each of the samples, the burial age was determined by dividing the paleodose by the environmental dose rate (Table S1). All ages are shown in ka relative to 2022, the first year of sample collection, with 1-σ symmetric uncertainty. Results are graphically shown in radial plots which indicate ages based on single-aliquot D_e estimates, and the final age estimate (Table S1) based on the environmental dose rate. The validity estimate in Table S1 is

based on a combination of the D_e distribution, the consistency of the ages obtained, and the luminescence properties of the samples. The results indicate that the deposits were formed between 2.40 ± 0.19 and 12.01 ± 0.77 ka.

Following the age calculations per individual sample, the median age and 1-σ standard deviation were calculated for the various geogenetic units (see Table 1), by stacking the individual Gaussian distribution curves of accepted OSL dating results within each unit where applicable. This approach weighs clustering of OSL ages, leaving the age of the particular unit less sensitive to outliers (14, 15). Table 1 provides all calculated median age results per unit.

Ceramic field-analysis. Ceramic pottery fragments were collected whenever encountered in the drilled sediments and were bagged in the field per 10 cm interval. After rinsing and drying, fragments were further analyzed and assigned, where possible, to their respective archaeological periods by means of typological dating (16), conforming to *Pancin* (17), *Salvador* (18) and *Welsby* (19). Assignments are based on the information from individual fragments combined with the assemblage of finds at the same level. A total of 118 sherds was retrieved with a minimum number of individuals (MNI) of 70 different vessels (Table S3). The ceramic fragments are mainly small body sherds, worn with rounded edges. What stood out is the overall composition of mainly everyday wares from storage, cooking, and table wares, but no clear fine wares. Samples with robustly assigned archaeological periods (i.e. Robustness: OK and Tentative; see Table S3) are shown in Fig. 2.

The indicated archaeological periods and the robustness estimate are expert judgement, based on extensive experience with the ceramic material itself, its texture, complexion and fabric (without freshly clipped breaks), wall thickness, possible shapes and functions and field photographs. Due to the severely deteriorated safety situation in Sudan as of this writing (October 2025), it was not possible to physically reevaluate any of the material; the identifications are largely based on field notes and photographs.

Radiocarbon dating. Two charcoal samples, originating from cultural layers in the upper central part of the East Mound (Fig. 2), were submitted to Beta Analytic Inc (Miami, Florida, USA) for radiocarbon dating using Accelerator Mass Spectrometry (AMS). The samples were previously calibrated with OxCal v4.3.2 (20) and the IntCal13 atmospheric curve (21) and published by *Emberling et al.* (2). The AMS dates correspond to the archaeological dating of the uncovered cultural remains. Note that these samples (Fig. S5) were retrieved from sieving of whole soil deposits: sample Barkal A17:10 comes from a deposit of approximately 4 by 4 m in extent and ca. 15 cm in depth; Barkal A27:5 comes from a deposit of approximately 3 by 2.5 m in extent and circa 30 cm in depth. In 2023, archaeological investigations on the East Mound uncovered Napatan period deposits at lower levels of the site. No samples from these lower deposits have yet been submitted for radiocarbon dating. See *Emberling et al.* (2) for full details.

For this study, the two 14C dates were recalibrated with OxCal v4.4.4 (22) using IntCal20 atmospheric data (23) (Fig. S6). An average uncalibrated date for the two samples of 2030 ± 21 14C yr BP was obtained with a weighted mean. Because the two samples are of the same material and layer, weighting was performed prior to calibration. The calibrated weighted mean result is 96 BC – 60 CE (2.12 – 1.96 ka), dating the cultural layers from the top of the East Mound to the Meroitic period (Table 1). To facilitate comparisons with both archaeological data and the OSL dating results, radiocarbon dating results are provided in uncalibrated ("14C") years BP, date

ranges in years calBP, date ranges in years calBCE/CE, and age ranges in ka relative to 2022 (the OSL reference year) (Table S5). All calibrated radiocarbon results are reported at 2-σ confidence.

CORONA satellite imagery. CORONA satellite imagery (24, 25) provides a historical snapshot of fluvial bar formation and vegetation establishment. The oldest bar, CB1a, fringes the T1 terrace remnant and reaches an elevation of ca. 250 m (Fig. 2A). This bar seemingly dates from just before 1968 CE, as CORONA imagery shows that its surface was still largely bare, with no large shrub-like vegetation at the time of recording. Westward, CB1b (ca. 249 m elevation) formed after 1968, as the CORONA imagery shows that this bar did not exist yet at the time of recording. Closest to the modern river, CB1c represents the youngest part of the Nile's channel belt and includes the active channel.

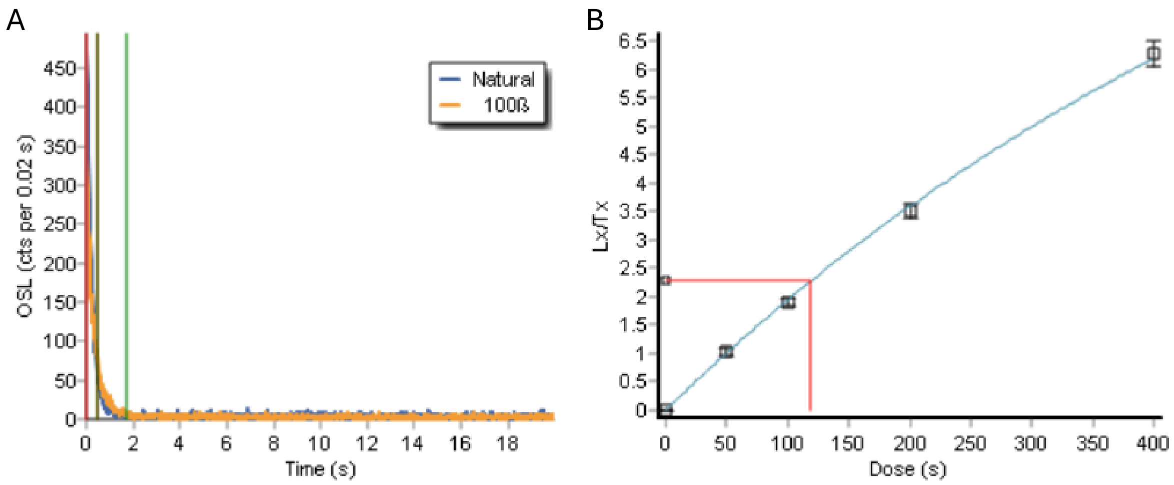

Figure S1. Example of typical OSL results including shine down (*A*) and dose response curve (*B*) for small-aliquot quartz of this project. These data are from sample CS011.D.

A

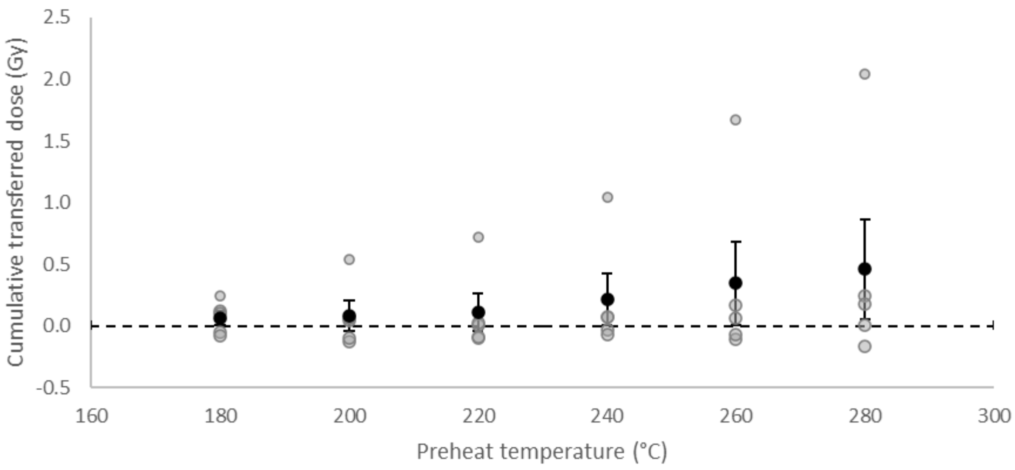

B

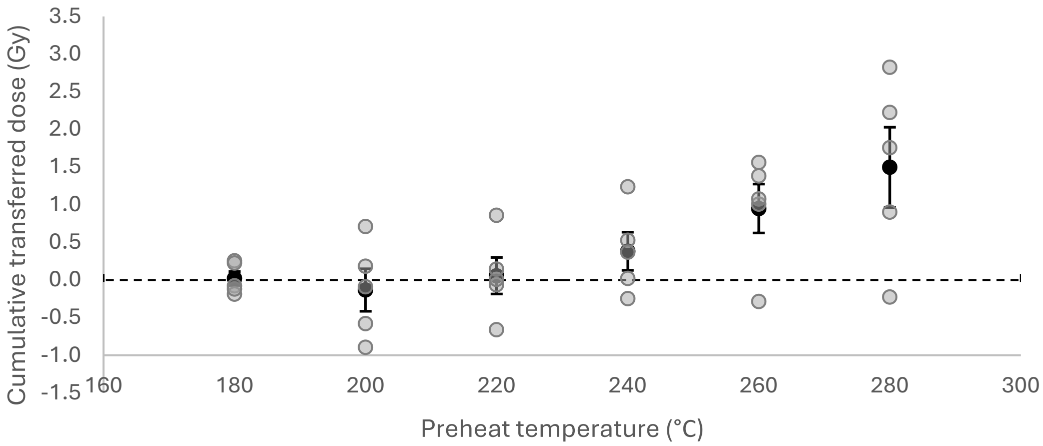

Figure S2. Thermal transfer test results for (*A*) sample CS016.B and (*B*) sample CS017.B.

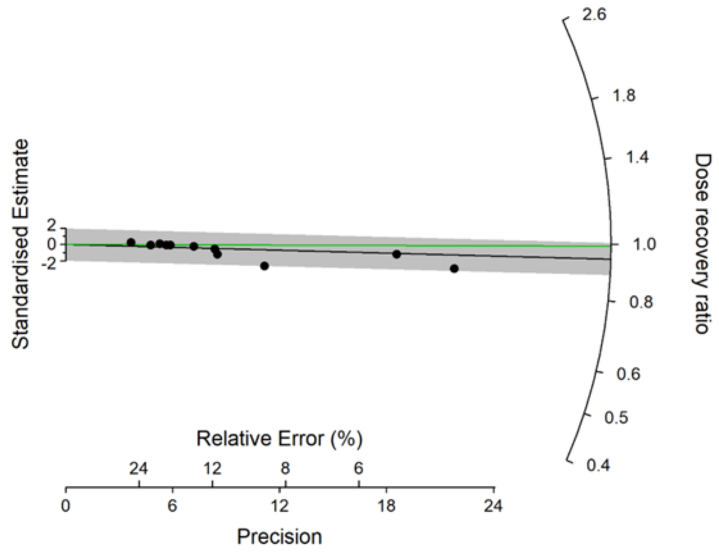

Figure S3. Radial plot showing measured dose recovery ratios for 11 accepted aliquots of NCL-7422 and NCL-7123. The iterated mean dose recovery ratio is 0.94 ± 0.02, indicated by the black line and grey shading. Unity is indicated by the green line.

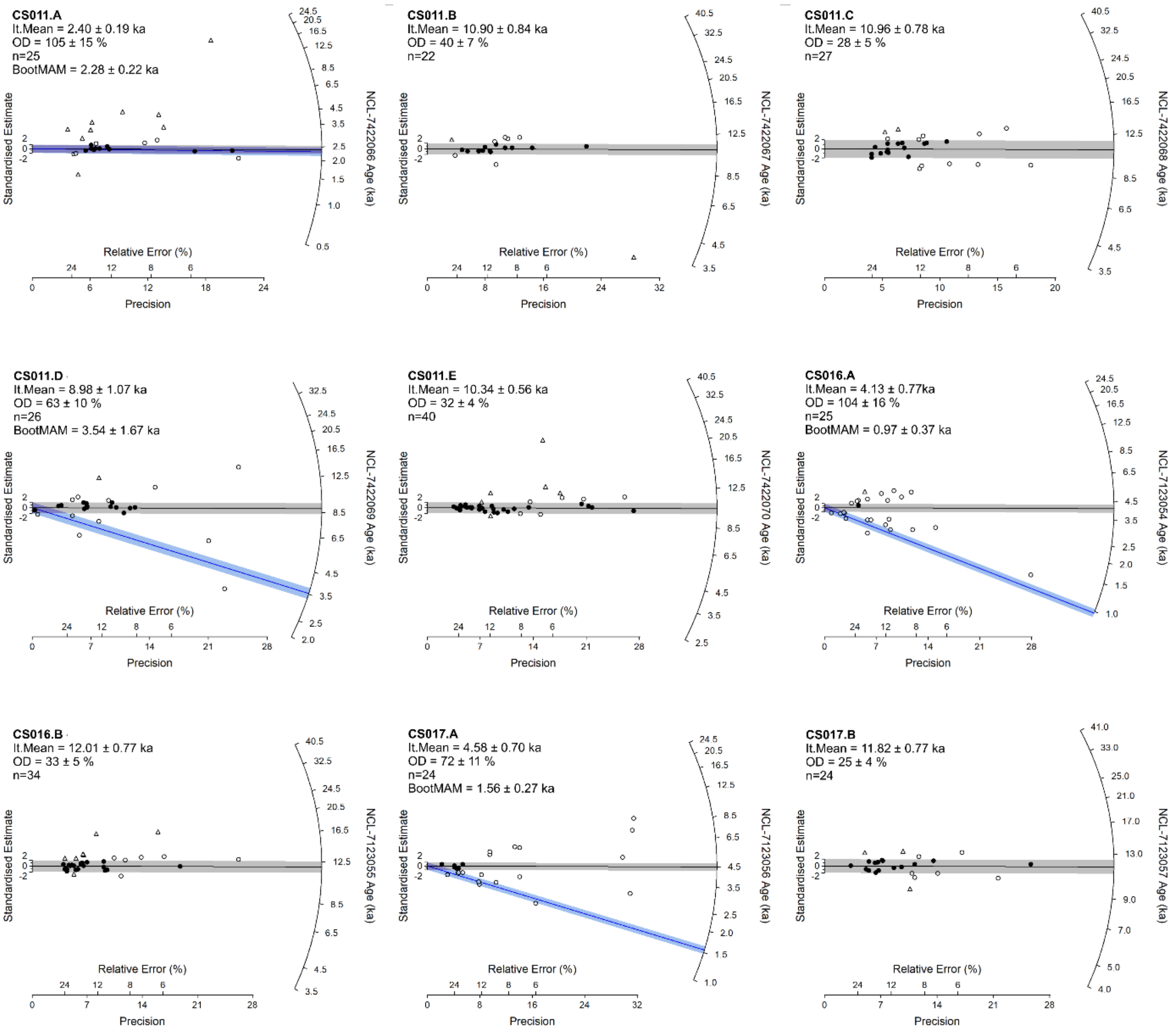

Figure S4. Radial plots showing age distributions for all samples. Single-aliquot OSL ages are indicated by open and filled dots; aliquots that were rejected by iteration are indicated by open triangles. The sample age obtained with the iterated mean is indicated by the black line, and the minimum age estimated with the bootMAM is indicated by the blue line (8, 26). The curved y-axis indicates the age estimate, whereas the x-axis reflects the precision of the individual estimates; the most well-known points plot on the right-hand side. To construct these graphs, single-aliquot palaeodose estimates were divided by the sample dose rate. Shading indicates the 2-σ uncertainty on the age model estimates. Aliquots in agreement with the iterated mean age are shown as filled black circles and aliquots in disagreement are shown as open circles. Uncertainties in dose rate and systematic uncertainties in paleodose estimation are not included in the graphs.

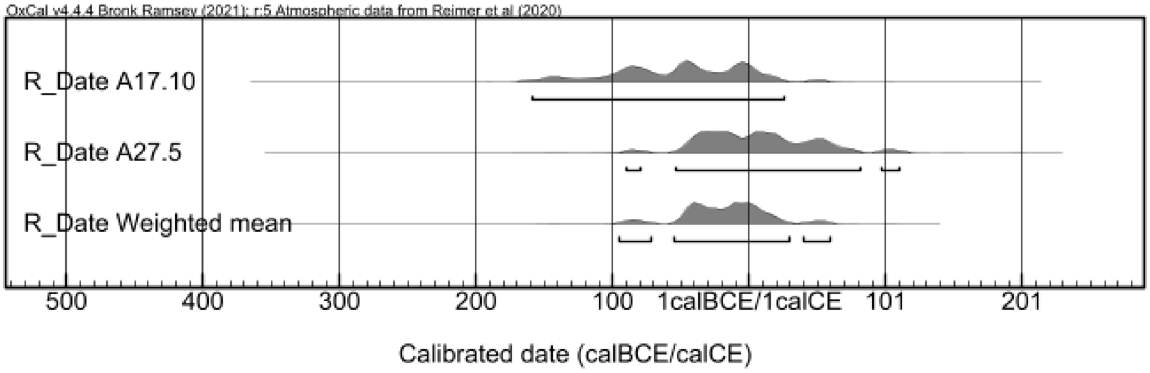

Figure S5. Timeline depicting the calibrated AMS radiocarbon dates from the East Mound Cultural layer. The dates are shown at 2-σ confidence in calendar years BCE/CE. Additional reporting formats can be found in Table S5. Calibration was performed using the IntCal20 calibration dataset (23); OxCal v4.4.4 was used creating this graph (22). Updated version of *Emberling et al.* (2).

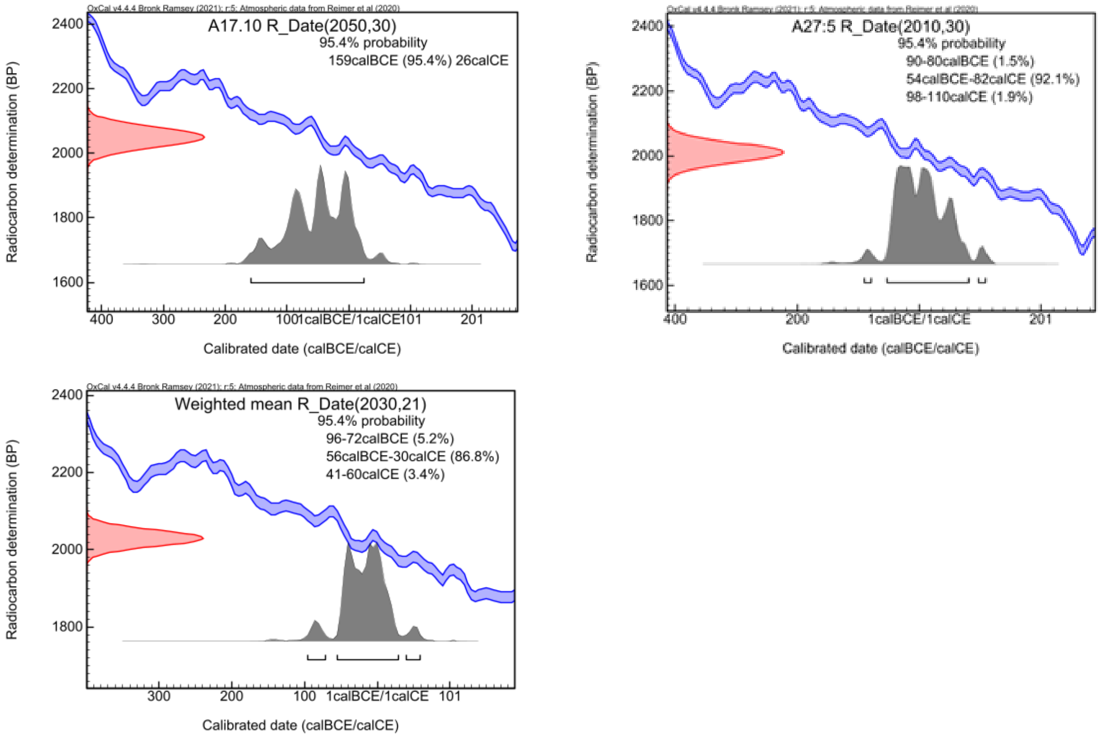

Figure S6. Calibration results of the radiocarbon dates. Including Barkal A17:10, Barkal A27:5, and a weighted mean of the two samples, performed in OxCal v4.4.4 (22) with the IntCal20 calibration set (23). The two-σ confidence intervals are shown in calibrated years BCE/CE. Updated version of *Emberling et al.* (2).

Table S1. OSL dating results

Sample name	NCL code	Depth (m)	Depositional environment	Burial regime	Aliquots accepted (n)	Alliquots after iteration (n)	Over-dispersion before iteration (%)	Over-dispersion after iteration (%)	Paledose (Gy)	Age model	Cosmogenic dose (Gy/ka)	U (Bq/kg)	Th (Bq/kg)	40K (Bq/kg)	Water content measured (% dry weight)	Water content assumed (% dry weight)	Organic content measured (% dry weight)	Organic content assumed (% dry weight)	Dose rate (Gy/ka)	Age (ka, 2022)	Validity
Core site CS011: 18°32'01"N, 31°49'49"E; 250.9 m																					
CS011.A	NCL-7422066	3.93-4.17	Floodplain	Gradual	25	16	105 ± 15	18 ± 5	4.27 ± 0.28<br>4.06 ± 0.34	Iterated mean<br>BootMAM (Sigma_b = 20±5%)	0.17 ± 0.01	22.5 ± 0.4	24.9 ± 0.8	338 ± 11	39.30	10.0 ± 5.0	2.41	2.0 ± 0.5	1.78 ± 0.09	2.40 ± 0.19<br>2.28 ± 0.22	Likely valid<br>Minimum age
CS011.B	NCL-7422067	4.93-5.17	Shallow terrace	Gradual	22	20	40 ± 7	22 ± 4	19.01 ± 1.14	Iterated mean	0.16 ± 0.01	19.8 ± 0.3	21.7 ± 0.7	346 ± 111	38.95	10.0 ± 5.0	2.15	2.0 ± 0.5	1.74 ± 0.09	10.90 ± 0.84	Valid
CS011.C	NCL-7422068	6.36-6.62	Deep terrace	Gradual	27	25	28 ± 5	23 ± 4	21.11 ± 1.19	Iterated mean	0.15 ± 0.01	21.4 ± 0.3	22.0 ± 0.7	442 ± 10	31.44	15.0 ± 5.0	1.34	2.0 ± 0.5	1.93 ± 0.09	10.96 ± 0.78	Valid
CS011.D	NCL-7422069	7.08-7.32	Deep terrace	Gradual	26	25	63 ± 10	57 ± 9	16.32 ± 1.80<br>6.44 ± 3.03	Iterated mean<br>BootMAM (Sigma_b = 20±5%)	0.15 ± 0.01	20.8 ± 0.3	22.0 ± 0.7	411 ± 9	30.12	15.0 ± 5.0	1.66	2.0 ± 0.5	1.82 ± 0.08	8.98 ± 1.07<br>3.54 ± 1.67	Likely valid<br>Minimum age
CS011.E	NCL-7422070	7.38-7.62	Deep terrace	Gradual	40	34	32 ± 4	10 ± 2	21.56 ± 0.67	Iterated mean	0.15 ± 0.01	22.9 ± 0.3	27.5 ± 0.9	466 ± 10	25.03	15.0 ± 5.0	1.23	2.0 ± 0.5	2.09 ± 0.09	10.34 ± 0.56	Valid
Core site CS016: 18°32'06"N, 31°50'01"E; 251.0 m																					
CS016.A	NCL-7123054	2.95-3.16	Floodplain	Gradual	25	24	104 ± 16	99 ± 15	7.35 ± 1.33<br>1.72 ± 0.66	Iterated mean<br>BootMAM (Sigma_b = 20±5%)	0.18 ± 0.01	18.5 ± 0.3	19.8 ± 0.7	397 ± 8	31.73	10.0 ± 5.0	7.11	5.0 ± 2.5	1.78 ± 0.08	4.13 ± 0.77<br>0.97 ± 0.37	Questionable<br>Minimum age
CS016.B	NCL-7123055	4.70-4.90	Shallow terrace	Gradual	34	27	33 ± 5	13 ± 3	25.93 ± 1.07	Iterated mean	0.17 ± 0.01	18.1 ± 0.3	18.0 ± 0.7	552 ± 11	28.33	10.0 ± 5.0	2.88	5.0 ± 2.5	2.16 ± 0.11	12.01 ± 0.77	Valid
Core site CS017: 18°32'05"N, 31°50'04"E; 251.6 m																					
CS017.A	NCL-7123056	5.79-6.00	Floodplain	Gradual	24	24	72 ± 11	72 ± 11	7.50 ± 1.09<br>2.55 ± 0.42	Iterated mean<br>BootMAM (Sigma_b = 20±5%)	0.16 ± 0.01	20.3 ± 0.2	24.6 ± 0.6	316 ± 6	40.55	10.0 ± 5.0	9.13	5.0 ± 2.5	1.64 ± 0.08	4.58 ± 0.70<br>1.56 ± 0.27	Questionable<br>Minimum age
CS017.B	NCL-7123057	8.84-9.04	Deep terrace	Gradual	24	21	25 ± 4	20 ± 4	21.83 ± 1.03	Iterated mean	0.14 ± 0.01	23.3 ± 0.2	30.0 ± 0.6	379 ± 7	28.84	15.0 ± 5.0	5.58	5.0 ± 2.5	1.85 ± 0.08	11.82 ± 0.77	Valid

Table S2. Single Aliquot Regenerative (SAR) procedure adopted for quartz

Step	Action	Measured
1	Natural dose or beta regenerative dose (Natural, 4 regen points up to 55 Gy, recuperation, recycling)	-
2	10 s preheat to 220 °C	-
3	20 s blue stimulation at 125 °C	L_n, L_i
4	Beta test dose	-
5	10 s cutheat to 200 °C	-
6	20 s blue stimulation at 125 °C	T_n, T_i
7	40 s blue bleach at 230 °C	-
8	Repeat step 1-7 for a range of doses	-
Extra 1*	Repeat step 1-7 with added infrared bleach at 30 °C prior to step 3	-

*Extra step 1 serves to check whether the sample is free of feldspar contamination (27).

Table S3. Summary of ceramic field-analysis of potsherds and their indicated archaeological periods

Year	Core site.sample number	Sample depth (m below surface)	Number of sherds	Minimum number of individuals (MNI)	Description of ceramic potsherds and function*	Other finds†	Archaeological period (indication)	Robustness	Edge appearance	Remarks
2022	CS001.01	1.90–2.00	5	1	5 BS. Probably from same vessel. Likely a storage jar.	-	Napatan	Tentative	Sub-rounded	-
2022	CS002.01	1.60–1.70	4	2	3 BS (WM).	1 RBF	Early Meroitic	OK	Rounded	-
2022	CS002.02	1.80–1.90	2	1	2 BS from closed jar (CM?).	-	Early Meroitic	OK	Sub-angular	-
2022	CS002.03	1.90–2.00	1	1	1 BS from jar (CM?).	-	Early Meroitic	OK	Sub-angular	-
2022	CS002.04	2.00–2.10	1	1	1 BS (HM?). Sort of tableware, closed vessel.	-	Meroitic?	Uncertain	Sub-rounded	Potential backfill
2022	CS002.05	2.90–3.00	1	1	1 BS (WM?). Thinner walled.	-	Napatan?	Uncertain	Rounded	-
2022	CS003.nn	-	-	-	-	-	-	-	-	No ceramic potsherds present
2022	CS004.01	3.70–3.80	1	1	1 BS.	-	Napatan?	Uncertain	Rounded	-
2022	CS005.01	5.70–5.80	1	1	1 BS. Completely fired through, rather orange, fabric appears more inorganic, sandy.	-	Napatan? Or older.	Uncertain	Rounded	-
2022	CS005.02	8.50–8.60	1	1	1 BS. Thin walled, dark red slip; pointing towards Napatan period.	-	Napatan? Or older.	Uncertain	Rounded	-
2022	CS006.01	4.70–4.80	1	1	1 BS (HM?).	-	Napatan	Tentative	Sub-rounded	-
2022	CS006.02	7.40–7.50	1	1	1 BS (HM?). Grooved, thin walled with a more pinkish fabric.	-	Napatan?	Uncertain	Rounded	-
2022	CS007.01	3.80–3.90	1	1	1 BS (WM?). Probably a bowl, dark red slip.	-	Napatan	Tentative	Rounded	-
2022	CS008.01	5.20–5.30	1	1	1 BS.	-	Napatan? Or older.	Uncertain	Rounded	-
2022	CS009.01	3.40–3.50	6	4	6 BS (mainly CM?). Probably storage jars; 1 BS (WM?). Tableware.	-	Napatan	Tentative	Rounded	-
2022	CS009.02	4.80–4.90	6	4	6 BS (CM). Storage jars.	-	Napatan	Tentative	Sub-rounded	-
2022	CS009.03	5.80–5.90	3	1	3 BS (CM?).	-	Napatan	Tentative	Sub-angular	-
2022	CS010.01	1.70–1.80	1	1	1 BS (WM). Bowl.	-	Early Meroitic	OK	Rounded	-
2022	CS010.02	2.00–2.10	1	1	1 BS (WM). Possibly from Krater-shaped vessel with a pink slip.	-	Early Meroitic	OK	Sub-rounded	-
2022	CS010.03	2.70–2.80	1	1	1 BS (WM). Likely a bowl.	1 STF	Early Meroitic	OK	Sub-rounded	-
2022	CS010.04	2.80–2.90	1	1	1 BS (WM).	-	Early Meroitic?	Uncertain	Rounded	-
2022	CS010.05	2.90–3.00	1	1	1 BS (WM).	-	Meroitic?	Uncertain	Rounded	-
2022	CS010.06	3.10–3.20	5	2	5 BS (WM); 1 BS (CM?). Bowl; 4 BS (CM?). Jar.	-	Napatan or transition to Meroitic	Tentative	Sub-rounded	-
2022	CS011.01	4.20–4.30	1	1	1 (WM). Rim of a simple bowl.	5 BF	Napatan? Or older.	Uncertain	Rounded	-
2022	CS012.nn	-	-	-	-	-	-	-	-	No ceramic potsherds present
2022	CS013.nn	-	-	-	-	-	-	-	-	No ceramic potsherds present
2022	CS014.nn	-	-	-	-	-	-	-	-	No ceramic potsherds present
2023	CS015.01	2.10–2.20	1	1	1 BS (WM/CM). Larger, probably open vessel.	-	Napatan or transition to Meroitic	Tentative	Rounded	-
2023	CS015.02	2.50–2.60	1	1	1 Rim (WM/CM). Larger open vessel, likely a storage jar.	-	Napatan or transition to Meroitic	Tentative	Sub-rounded	-
2023	CS016.01	0.70–0.80	3	2	3 BS (WM).	-	Early Meroitic	Tentative	Sub-angular	-

Table S3. (*continued*)

2023	CS016.02	0.90–1.00	1	1	1 base (CM). Thick-walled, crude executed storage jar.	-	Early Meroitic	Tentative	Sub-angular	-
2023	CS016.03	1.20–1.30	1	1	1 BS (HM).	-	Napatan or transition to Meroitic	Tentative	Rounded	Very worn
2023	CS016.04	1.70–1.80	1	1	1 BS (HM).	-	Napatan or transition to Meroitic	Tentative	Sub-rounded	Very worn
2023	CS016.05	2.00–2.10	20	3	20 BS; 18 BS (HM). 2 BS (IMP) or Imitation. Napatan jar.	1 BF	Napatan	OK	Sub-angular	Potential backfill
2023	CS017.01	0.20–0.30	2	1	2 BS (CM). Storage jar.	-	Meroitic	OK	Sub-angular	-
2023	CS017.02	0.50–0.60	1	1	1 BS (WM). Bowl.	13 BF	Early Meroitic	Tentative	Angular	-
2023	CS017.03	0.60–0.70	1	1	1 BS (HM).	-	Napatan or transition to Meroitic	Tentative	Sub-angular	-
2023	CS017.04	0.70–0.80	4	2	4 BS (HM).	2 BF	Late Napatan?	Uncertain	Sub-angular	-
2023	CS017.05	1.00–1.10	2	1	2 Rims (HM). Bowl.	-	Late Napatan?	Uncertain	Sub-angular	-
2023	CS017.06	1.20–1.30	2	2	2 BS (HM).	1 BF	Late Napatan?	Uncertain	Sub-rounded	-
2023	CS017.07	1.60–1.70	8	2	8 BS (HM).	-	Late Napatan?	Uncertain	Sub-angular	-
2023	CS017.08	2.20–2.30	-	-	-	1 BF	-	-	-	No ceramic potsherds present
2023	CS017.09	2.80–2.90	1	1	1 BS. Possibly an IMP or Imitation.	2 BF	Napatan?	Uncertain	Sub-angular	-
2023	CS018.01	0.70–0.80	4	3	4 BS (HM). 2 fragments could possibly be part of a cooking installation, complete burned on lower site, there seems to be a burnish or polish on the upper site, all rather brown colored.	-	Napatan or transition to Meroitic?	Uncertain	Sub-rounded	-
2023	CS018.02	1.10–1.20	1	1	1 BS (WM/IMP or Imitation).	2 STF	Napatan/Meroitic?	Uncertain	Sub-rounded	-
2023	CS018.03	1.70–1.80	1	1	1 BS (HM). Thin walled.	-	Napatan/Meroitic?	Uncertain	Sub-angular	
2023	CS018.04	4.30–4.40	-	-	-	1 BF	-	-	-	No ceramic potsherds present
2023	CS019.01	1.50–1.60	3	2	1 Rim (WM). Small bowl; 2 BS (CM). Larger jars.	-	Napatan	Tentative	Sub-angular	-
2023	CS020.01	0.50–0.60	1	1	1 BS (HM).	-	Meroitic?	Uncertain	Rounded	-
2023	CS020.02	2.50–2.60	1	1	1 BS (CM). Thick dark red slip internal.	-	Napatan?	Uncertain	Rounded	-
2023	CS020.03	2.80–2.90	1	1	1 BS (WM). Tableware, likely a bowl, dark red slip external, brownish at the interior.	-	Napatan?	Uncertain	Sub-rounded	-
2023	CS021.01	0.70–0.80	2	2	2 BS (WM). Could be part of a burner.	-	Early Meroitic	Tentative	Sub-rounded	-
2023	CS021.02	1.00–1.10	4	2	4 BS (IMP). Possibly Aswan ware or Imitation. One vessel unit seems to have a cream marl clay fabric.	-	Napatan	Tentative	Sub-angular	-
2023	CS021.03	1.30–1.40	1	1	1 BS (HM).	1 STF	Napatan?	Uncertain	Rounded	-
2023	CS021.04	2.10–2.20	2	2	2 BS (HM?).	-	Napatan	Tentative	Sub-angular	-
2023	CS022.01	3.50–3.60	1	1	1 BS (HM). Fabric / complexion appears different.	-	Napatan? Or older.	Uncertain	Sub-angular	-
2023	CS023.nn	-	-	-	-	-	-	-	-	No ceramic potsherds present
2023	CS024.nn	-	-	-	-	-	-	-	-	No ceramic potsherds present
2023	CS025.nn	-	-	-	-	-	-	-	-	No ceramic potsherds present
2023	CS026.nn	-	-	-	-	-	-	-	-	No ceramic potsherds present

* Abbreviations: BS: Body sherd; WM: Wheel made; HM: Handmade; CM: Composite vessel; IMP: Import vessel.
† Abbreviations: BF: Bone fragment; RBF: Red brick fragment; STF: Stone fragment.

Table S4. Cultural periods at Jebel Barkal

Period	Abbreviation	Age[§]	Age (ka)	Reference
Islamic	Isl	1400–present	0.6–present	Edwards (28); Daly and Kaye (29)
Medieval (Christian)	Med	550–1400 CE	1.5–0.6	Edwards (28); Daly and Kaye (29)
Post-Meroitic	PM	350–550 CE	1.7–1.5	Edwards (28)
Meroitic	Mer	270 BCE–350 CE	2.3–1.7	Edwards (28); Török (30)
Napatan	Nap	1070–270 BCE	3.1–2.3	Edwards (28); Török (30)
New Kingdom domination	NK	1500–1070 BCE	3.5–3.1	Edwards (28); Hornung (31)
Kerma	n/a	2500–1500 BCE	4.5–3.5	Edwards (28); Honegger (32)
Pre-Kerma	PK	3500–2500 BCE	5.5–4.5	Honegger (32)
Neolithic	n/a	6000–3500 BCE	8.0–5.5	Edwards (28); Usai (33)
Mesolithic / Epipalaeolithic	n/a	10,000–6000 BCE	12.0–8.0	Edwards (28); Usai (33)

[§]Ages for all periods are approximate and may vary by up to several centuries due to limited chronological resolution.

Table S5. AMS radiocarbon dates from the East Mound Cultural layer at Jebel Barkal§

Sample name	Laboratory number	14C age (yr BP)	Calibrated age range, 2-σ (yr calBP)	Calibrated age range, 2-σ (yr calBCE/CE)	Calibrated age range (ka, 2022)
Barkal A17:10	Beta-521729	2050 ± 30	2108–1925 (95.4%)	159 BCE–26 CE (95.4%)	2.18–2.00
Barkal A27:5	Beta-521730	2010 ± 30	2039–2029 (1.5%)<br>2003–1869 (92.1%)<br>1852–1840 (1.9%)	90–80 BCE (1.5%)<br>54 BCE–82 CE (92.1%)<br>98 CE–110 CE (1.9%)	2.11–1.91
Weighted mean	n/a	2030 ± 21	2045–2021 (5.2%)<br>2005–1920 (86.8%)<br>1910–890 (3.4%)	96 BCE–72 BCE (5.2%)<br>56 BCE–30 CE (86.8%)<br>41 CE–60 CE (3.4%)	2.12–1.96

§Data obtained from radiocarbon samples previously published by *Emberling et al. (2024)*. The table provides the sample name, laboratory number at Beta Analytic, uncalibrated date in 14C years BP, date range in years calBP, date range in years calBCE/CE, and age range in ka relative to 2022 (the OSL reference year). All ages are reported at 2-σ confidence. Note that BP is referenced to 1950.

Dataset S1 (separate file). Sediment core dataset Jebel Barkal.

SI References

1. C. Ditzler, K. Scheffe, H.C. Monger, Eds., *Soil Survey Manual* (U.S. Dept. Agriculture, 2017).
2. G. Emberling *et al.*, Jebel Barkal 2018–2023: new research on the Napatan and Meroitic city. *Sudan & Nubia* **28**, 73–98 (2024).
3. A.G. Wintle, Luminescence dating: Laboratory procedures and protocols. *Radiation Measurements* **27**, 769–817 (1997).
4. N. Porat, Use of magnetic separation for purifying quartz for luminescence dating. *Ancient TL* **24**, 33–36 (2006).
5. A.S. Murray, A.G. Wintle, The single aliquot regenerative dose protocol: potential for improvements in reliability. *Radiation Measurements* **37**, 377–381 (2003).
6. A.C. Cunningham, J. Wallinga, Selection of integration time intervals for quartz OSL decay curves. *Quaternary Geochronology* **5**, 657–666 (2010).
7. G.A.T. Duller, Distinguishing quartz and feldspar in single grain luminescence measurements. *Radiation Measurements* **37**,161–165 (2003).
8. A.C. Cunningham, J. Wallinga, Realizing the potential of fluvial archives using robust OSL chronologies. *Quaternary Geochronology* **12**, 98–106 (2012).
9. R.F. Galbraith, R.G. Roberts, G.M. Laslett, H. Yoshida, J.M. Olley, Optical dating of single and multiple grains of quartz from Jinmium rock shelter, northern Australia: Part I, experimental design and statistical models. *Archaeometry* **41,** 339–364 (1999).
10. D. Vandenberghe, F. De Corte, J.-P. Buylaert, J. Kučera, On the internal radioactivity in quartz. *Radiation Measurements* **43**, 771–775 (2008).
11. J.R. Prescott, J.T. Hutton, Cosmic-ray contributions to dose-rates for luminescence and ESR dating — large depths and long-term time variations. *Radiation Measurements* **23**, 497–500 (1994).
12. V. Mejdahl, Thermoluminescence dating — beta-dose attenuation in quartz grains. *Archaeometry* **21**, 61–72 (1979).
13. M.J. Aitken, *An introduction to optical dating: the dating of Quaternary sediments by the use of photon-stimulated luminescence* (Oxford University Press, 1998).
14. J. Peeters *et al.*, Shift away from Nile incision at Luxor ~4000 years ago impacted ancient Egyptian landscapes. *Nat. Geosci.* **17**, 645–653 (2024).
15. J. Peeters *et al.*, Sedimentary architecture and chronostratigraphy of a late Quaternary incised-valley fill: a case study of the late Middle and Late Pleistocene Rhine system in the Netherlands. *Quat. Sci. Rev.* **131**, 211–236 (2016).
16. R. David, Ed., *Concise Manual for Ceramic Studies: from the Nile Valley to the Middle East.* Études d'Égyptologie **24,** (Soleb, 2022).
17. F. Pancin, "Meroitic pottery classification at Jebel Barkal" in *Jebel Barkal — Half a century of the Italian Archaeological Mission in Sudan*, E. Ciampini, F. Innarilli, Eds. (Gangemi Editore, 2022), pp.135–142.
18. A. Salvador, "The Meroitic pottery in Napata" in *Jebel Barkal — Half a century of the Italian Archaeological Mission in Sudan*, E. Ciampini, F. Innarilli, Eds. (Gangemi Editore, 2022), pp. 129–134.

19. I. Welsby Sjöström, *Gematon: Living and dying in a Kushite town on the Nile*. Volume 3, The pottery, (Sudan Archaeological Research Society, 2023).
20. C. Bronk Ramsey, OxCal v4.3.2. Oxford Radiocarbon Accelerator Unit, University of Oxford, United Kingdom (2017). https://c14.arch.ox.ac.uk/oxcal.html. Accessed 14 October 2025.
21. P.J. Reimer *et al.*, INTCAL13 and MARINE13 radiocarbon age calibration curves 0–50,000 years CAL BP. *Radiocarbon* **55**, 1869–1887 (2013).
22. C. Bronk Ramsey, OxCal v4.4.4. Oxford Radiocarbon Accelerator Unit, University of Oxford, United Kingdom (2021). https://c14.arch.ox.ac.uk/oxcal.html. Accessed 14 October 2025.
23. P.J. Reimer *et al.*, The INTCAL20 Northern Hemisphere radiocarbon age calibration curve (0–55 CAL kBP). *Radiocarbon* **62**, 725–757 (2020).
24. A. Dashora, B. Lohani, J.N. Malik, A repository of earth resource information – CORONA satellite programme. *Current Science* **92**, 926–932 (2007).
25. Center for Advanced Spatial Technologies (CAST), Corona Atlas & Referencing System, Corona image: 1105–2235F112 [18 November 1968]. University of Arkansas, United States Geological Survey, USA. Available at: https://corona.cast.uark.edu/. Accessed on 14 October 2025.
26. R.F. Galbraith, The radial plot: graphical assessment of spread in ages. *International Journal of Radiation Applications and Instrumentation. Part D. Nuclear Tracks and Radiation Measurements* **17**, 207–214 (1990).
27. A.G. Wintle, A.S. Murray, A review of quartz optically stimulated luminescence characteristics and their relevance in single-aliquot regeneration dating protocols. *Radiation Measurements* **41**, 369–391 (2006).
28. D. Edwards, *The Nubian past: an archaeology of Sudan* (Routledge, London, 2004).
29. M.W. Daly, A.S. Kaye, “Sūdān” in *Encyclopaedia of Islam New Edition Online*, P. Bearman, (Ed.) (Brill, Leiden, 2012). https://referenceworks.brill.com/display/db/eieo. Accessed 23 June 2025.
30. L. Török, The Kingdom of Kush, *Handbook of the Napatan–Meroitic Civilization,* (Brill, Leiden, 1997).
31. E. Hornung, R. Krauss, D.A. Warburton (Eds.), Ancient Egyptian Chronology. *Handbook of Oriental Studies,* (Brill, Leiden, 2006).
32. M. Honegger, “The Pre-Kerma Culture and the Beginning of the Kerma Kingdom” in *The Oxford handbook of Ancient Nubia*, G. Emberling, B.B. Williams (Eds.) (Oxford University Press, 2020), pp. 143–156.
33. D. Usai, A Picture of Prehistoric Sudan: The Mesolithic and Neolithic Periods, *The Oxford Handbook of Topics in Archaeology*, Oxford Handbooks Editorial Board (online edition, Oxford Academic Press, 2014). https://doi.org/10.1093/oxfordhb/9780199935413.001.0001. Accessed 24 June 2025.
